# Prevalence and influencing factors of co-morbid depression in patients with type 2 diabetes mellitus: a General Hospital based study

**DOI:** 10.1186/s13098-015-0053-0

**Published:** 2015-06-30

**Authors:** Weijun Zhang, Huiwen Xu, Shuliang Zhao, Shinan Yin, Xiaohua Wang, Jing Guo, Shengfa Zhang, Huixuan Zhou, Fugang Wang, Linni Gu, Lei Zhu, Haibo Yu, Zhiyong Qu, Donghua Tian

**Affiliations:** School of Social Development and Public Policy, China Institute of Health, Beijing Normal University, 19, Xinjiekou Wai Street, Beijing, 100875 China; Department of Public Health Sciences, University of Rochester School of Medicine & Dentistry, Rochester, NY 14642 USA; School of Public Administration, Yunnan University of Finance and Economics, Kunming, 650221 China; Department of Endocrinology, First Affiliated Hospital of the General Hospital of the People’s Liberation Army (PLA), Beijing, 100853 China; Department of Sociology, Huazhong University of Science and Technology, 1037 Luoyu Road, Wuhan, 430074 China; School of Government, Beijing Normal University, 19, Xinjiekou Wai Street, Beijing, 100875 China

**Keywords:** Type 2 diabetes mellitus, Depression, Prevalence, Risk factors

## Abstract

**Background:**

Depression and diabetes have been recognized as major public health issues in China, however, no studies to date examined the factors associated with the development of depression in patients with diabetes in China. This study aimed to estimate the prevalence of co-morbid depression among adults with type 2 diabetes mellitus (DM) and to examine the influence factors of co-morbid depression in a group of patients with type 2 DM.

**Methods:**

The study was conducted from March l to May 31, 2012, in the Department of Endocrinology of the First Affiliated Hospital of the General Hospital of the People’s Liberation Army (PLA). A systematic random sample of 412 type 2 DM patients aged over 18 years was selected. A structured questionnaire was used for collecting the information about socio-demographic data, lifestyle factors and clinical characteristics. Depression and social support was evaluated by using the Chinese version of Beck Depression Inventory (BDI) and Social Support Rate Scale (SSRS), respectively. Weights and heights were measured. Hemoglobin A1c (HbA1c) was abstracted from each patient directly after the interview.

**Results:**

Of the total sample, 142 patients had depression according to the BDI scores (BDI scores ≥14), the prevalence of co-morbid depression in this study population was 5.7 % (142/2500). Of which, 56 had major depression (BDI ≥ 21), and 86 had moderate depression (BDI ≥ 14&BDI < 21). Logistic regression analysis indicated that a high HbA1c level, a high BMI, low quality health insurance, and being single, were significantly associated with the development of depression. However, a family history of diabetes and a high social support level are likely protective factors.

**Conclusions:**

The prevalence of co-morbid depression was 5.7 % among Chinese subjects with type 2 DM in this study. High HbA1c level, high BMI score, being single, low social support level, and low quality health insurance were associated with the presence of depression. These findings support a recommendation for routine screening and management in China for depression in patients with diabetes, especially for those in primary care, to reduce the number of the depressed or the misrecognized depressed diabetic patients.

**Electronic supplementary material:**

The online version of this article (doi:10.1186/s13098-015-0053-0) contains supplementary material, which is available to authorized users.

## Introduction

Diabetes was one of the most important public health problems in China, and there were 113.9 million patients with diabetes and 493.4 million patients with pre-diabetes among Chinese adults in 2010 [1]. There was also evidence that confirms the adverse economic impacts of co-morbid diabetes and depression [2]. The direct medical costs of type 2 DM and its complications were projected to be 47.2 billion USD in 2030 [3]. Meanwhile, the total estimated cost of depression in China was 6264 million USD at 2002 prices [4]. Previous studies suggested that the prevalence of depressive disorders and its’ risk factors may differ between countries and within countries, and across various ethnicities [5, 6]. It may be related to the differences in either the presentation of symptoms or a lack of cultural appropriateness of western methods of identifying depression [7].

Evidence suggested that diabetes and depression could mutually exacerbate, with each condition acting as a risk factor in the development of the other [8–10]. Songar et al. used a Beck’s Depression Inventory score of 14 as cut-off and studied 60 diabetic patients and 30 healthy controls, and found a prevalence of 43.3 % and 3.3 % respectively [11]. In another study, conducted by Peyrot and Rubin, a prevalence of 41.3 % was found among 634 diabetic patients, by using CES-D 16 as cut-off [12]. Pouwer et al. presented a prevalence of 32 % from a random sample of 772 mixed cases of diabetes for study, by using the composite international diagnostic interview and CES-D (cut-off 16) [13]. Raval et al. reported a prevalence of 41 % in North India by using the Patient Health Questionnaire (PHQ)-9 scale [14], while Poongathai et al. reported only 23.4 % from the South of India, by using the PHQ-12 [15]. In 2014, Singh H et al. found a CES-D defined depression prevalence of 42.2 % in diabetes patients in india, which is 10 times higher than controls (4.39 %) [16]. In a meta-analysis of 10 randomly controlled trials on type 2 diabetes mellitus (DM) only, a significantly higher prevalence of depression was found in people with diabetes compared with those without (17.6 vs. 9.8 %, OR = 1.6, 95 % CI 1.2–2.0) [17]. A recent systematic review indicated that individuals with previously diagnosed diabetes have an increased risk of depression compared to those with impaired glucose metabolism or undiagnosed diabetes (OR 1.69, 95 % CI 1.37–2.08) [18]. In addition, other studies indicated that type 2 DM was a risk factor for developing depression [19, 20], the incidence of depression was 1.8-times higher in the diabetic group than in nondiabetic subjects over a median follow-up of 6.5 years [21].

Meanwhile, the prevalence of depression always varies internationally among different populations [22]. The higher depression prevalence was found in African Americans with diabetes than in their counterparts of white northern European ancestry [23, 24]. Even higher levels of co-morbid diabetes and depression were also shown in the US Latino population [25–28], Native Americans [29], and Palestine [30]. Overall, it is now generally agreed that the prevalence of depression increased in people with diabetes, although the study, on the Pima Indians in Arizona, found no significant differences in depressive symptoms between those with and without diabetes [31]. Extensive researches had examined the risk factors of depression in patients with type 2 DM, the results varied widely across studies [22]. Depression risk factors that are specific to diabetes, included co-morbidity of diabetes-related complications [32], macro-vascular disease (stroke, peripheral artery disease) [33], micro-vascular disease including retinopathy [34], neuropathy and nephropathy [35], longer duration of diabetes [36, 37], more demanding regimens [38], negative life events [39], low levels of daily activities [40, 41], physical activity [42], perceived burden of diabetes [43], diabetes diet, increased cholesterol and difficulty in daily living [33], being a female, and being widowed or divorced [44]. Besides, the depression symptoms was also significantly associated with poorer glycemic control [45, 44, 46, 39], the level of HbA1c [45], high fasting insulin values, 2-h glucose concentrations and insulin resistance [47]. However, several studies showed that the relationship between depression and glycemic control/impaired fasting glucose was uncertain [48] or insignificantly [49–51]. Another 5-year follow-up study showed that previous depression history, baseline diabetes symptoms, and having had cardiovascular procedures significantly predicted major depressive disorder [34]. The course of depression in patients with diabetes is chronic and severe [52], and has significant effects on the outcome of their medical illness [53], including lower quality of life [54], poorer diabetes self-care [55], and an increased risk of developing diabetes-related complications [32]. Just because of this, *the International Diabetes Federation (IDF)* and *National Institute for Health and Clinical Excellence (NICE)* recommended that all patients with diabetes should undergo a regular screening for depression [56, 57].

To date, no studies have examined the factors associated with depression in patients with diabetes in China. This study aims to investigate the prevalence of depression and explore the influence factors associated with depression in a group of patients with type 2 DM.

## Methods

### Participants

The study was conducted from March l to May 31, 2012, in the Department of Endocrinology of the First Affiliated Hospital of the General Hospital of the People’s Liberation Army (PLA), which is one of the best medical centers in China. All of the patients above 18 years old who were diagnosed with type 2 DM according to the diagnostic criteria of the 10th revision of the International Classification of Diseases (ICD-10) [58], were selected from their medical records. The excluded patients were those having a history of psychiatric illness and those taking anti-depressant treatment and/or using psychotropic drugs, considering that antidepressants or psychotropic drugs may influence the outcome of BDI test. Eventually, there were 12 patients excluded from this study, including 3 patients having a history of psychiatric illness other than depression and 9 patients taking anti-depressant treatment, according to the medical records. 450 subjects were recruited by circular systematic random sampling into this study from 2500 patients. Specifically, the number of 135 was selected as the random starting point, and 6 was taken as the sampling interval. All of the patients were approached at the hospital by their attending diabetologist or diabetes nurse. Five diabetologists and 10 nurses were in charge of completing a structured questionnaire, including demographic information, BDI-21 test, and Social Support Rate Scale (SSRS), measuring weight and heights, and collecting the blood sample of subjects, in a hospital waiting room. The whole process took about 20–30 min per subject. An instruction and scoring manual was supplied to each diabetologist and specialized diabetes nurse in this study. The study protocol was approved by the School of Social Development and Public Policy (SSDPP) ethics committee of Beijing Normal University (BNU) and the research oversight committee of the General Hospital of PLA. The patients were provided written informed consents before the interview.

### Measurements

#### Demographic information

The eligible subjects were interviewed using a structured questionnaire. The demographic and socioeconomic factors including age, gender, education level, marriage status, and lifestyle factors (e.g., exercise times per week) were collected.

#### Depression

The depressive symptoms were assessed with the Chinese version of the self-administered 21-item Beck Depression Inventory (BDI) [59], and a cut-off value ≥14 was used to define the cases with symptoms of depression in the Chinese population. Cronbach’s α of the BDI in this study was 0.88.

#### Social support

Social support was evaluated by using the 14-item social support rate scale (SSRS), which measures objective support, subjective support, and useless support; it was developed by Xiao Shuiyuan in 1986 and validated in a previous study [60]. Cronbach’s alpha of the SSRS in this sample was 0.74, the maximum scores were 50 and the minimum scores were 20, respectively.

#### Clinical characteristics

Glycated Haemoglobin (HbA1c), which is used to measure blood glucose control over several months and provides an estimate of how well diabetes has been controlled over the last 2 or 3 months, was abstracted from each patient directly after the interview. HbA1c determination for all samples was done in the same laboratory by the same technician, who was blind to the diagnosis results of the patients, and applying the same technique. Weight and heights were also measured to calculate the Body Mass Index (BMI). Other clinical characteristics including disease duration, diabetes family history, and diabetic complications (hypertension, cardiac disease, diabetic nephropathy, diabetic retinopathy, and diabetic peripheral angiopathy) were obtained from the standard medical records of the patients under the permission of charge doctors.

### Statistical analysis

The descriptive statistics were used in accordance with the level of the variable measurements. The odds ratio (OR) and its 95 % confidence intervals (CIs) were estimated. The statistical significance of associations was evaluated using the *chi*-square test, and the *t* test was performed as appropriate. The level of statistical significance was fixed at 0.05 for the bi-marginal null hypothesis. Multivariate logistic regression analysis were applied to explore the factors associated with depression symptoms. The depression scores (≥14 versus <14) were used as the dependent variable and the independent variables including the demographic factors, clinical factors, and behaviour factors were introduced into the multivariate logistic regression model, stepwise. All the data were analysed by using SPSS 17.0 (SPSS Inc., Chicago, IL, USA), and *p* value of < 0.05 was considered statistically significant.

## Results

### Demographic characteristics

412 patients participated in this study successfully, for a response rate of 91.6 %. The other 38 patients, including 18 males, explicitly declined participation in the screening when they were approached by a nurse in the waiting room. These 38 patients did not differ in the demographic variables and the part clinical variables compared with the respondents (Additional file 1). The average age of the sample was 59.77 years (SD 12.48), with a range of 25 to 89 years; 53.6 % of the sample were over 60, and females comprised 49.8 % of the sample. For all the respondents, 70.1 % were married or living with a partner, and 29.9 % were single (including unmarried, divorced and widowed). Of the total sample, 13.3 % had a primary education (≤6 years), 39.5 % had completed junior middle school/high school (6–12 years), and 47.1 % had a junior college or more advanced education. Regarding employment, a high percentage of the subjects was retired (62.1 %), followed by being employed (33.5 %) and being unemployed (4.4 %) (Table 1).

**Table 1:** Co-morbid depression (BDI score ≥ 14) and demographic, clinical, and behavior characteristics by depression groups

Variables	Total sample (n)	No depression n(%)	Depression n(%)	Significance
n(%)	412	270(65.53)	142(34.47)	
Demographic factors				
Men	207(50.24)	134(49.63)	73(51.41)	(χ^2^ = 0.12;*P* = 0.73)
Female	205 (49.76)	136(66.3)	69(33.7)	
Age (years)				
<40	26(6.31)	9(3.33)	17(11.97)	(χ^2^ = 21.51; *P* = 0.00)
40–59	165(40.05)	126(46.67)	39(27.46)	
≥60	221(53.64)	135(50.00)	86(60.56)	
High school or less	218(52.91)	133(49.26)	85(59.86)	(χ^2^ = 4.20; *P* = 0.04)
Being ingle	123(29.85)	48(17.78)	75(52.82)	(χ^2^ = 54.55; *P* = 0.00)
Employment status				
Employed	138(33.50)	94(34.81)	44(30.99)	(χ^2^ = 6.09; *P* = 0.048)
Unemployed	18(4.37)	7(2.59)	11(7.75)	
Retired	256(62.14)	169(62.59)	87(61.27)	
Insurance				
GIS	130(31.55)	99(36.67)	31(21.83)	(χ^2^ = 22.88; *P* = 0.00)
UEMI	112(27.18)	68(25.19)	44(30.99)	
URMI	102(24.76)	60(22.22)	42(29.58)	
NCMS	17(4.13)	16(5.93)	1(0.70)	
Other insurance	19(4.61)	7(2.59)	12(8.45)	
None insurance	32(7.77)	20(7.41)	12(8.45)	
Social support	31.35 ± 5.50	33.45 ± 4.53	27.35 ± 4.94	(t = 12.60; *P* = 0.00)
Clinical factors				
HbA1c level				
<7.00 %	209(50.73)	152(56.30)	57(40.14)	(χ^2^ = 9.72; *P* = 0.002)
≥7.00 %	203(49.27)	118(43.70)	85(59.86)	
BMI(kg/m^2^)				
<25	215(52.18)	142(52.59)	73(51.41)	(χ^2^ = 0.05; *P* = 0.0819)
≥25	197(47.82)	128(47.41)	69(48.59)	
History of diabetes	190(46.12)	144(53.33)	46(32.39)	(χ^2^ = 16.42; *P* = 0.00)
Duration of diabetes in years	8.93 ± 6.51	8.20 ± 5.79	10.32 ± 7.52	(t = −3.17; *P* = 0.002)
Diabetes complications	2.39 ± 1.90	2.06 ± 1.58	3.01 ± 2.28	(t = −4.98; *P* = 0.00)
Oral agents	227(55.10)	148(55.64)	79(54.11)	(χ^2^ = 0.137; *P* = 0.76)
Insulin	185(44.90)	118(44.36)	67(45.89)	
Behavior factors				
Smoking currently	112(27.18)	80(29.63)	32(22.54)	(χ^2^ = 2.37; *P* = 0.12)
Drinking currently	198(48.06)	143(52.96)	55(38.73)	(χ^2^ = 7.55; *P* = 0.006)
Excise times per week	1.67 ± 2.78	2.00 ± 2.79	1.04 ± 2.67	(t = 3.35; *P* = 0.00)
Sleeping hours per day	5.95 ± 2.82	6.38 ± 1.22	5.13 ± 4.39	(t = 4.38; *P* = 0.00)

### Clinical characteristics and frequency of depression

Of the total sample, the average duration of diabetes was 8.93 years (SD 6.51), ranging from 1–36 years. Specifically, the average duration of diabetes for the patients with depression symptoms was longer than that without depression symptoms (10.32 ± 7.52 *vs* 8.20 ± 5.79, *t* = −3.17; *P* = 0.002). In addition, 83.3 % of the sample had diabetes complications, and the most frequently diagnosed complications were hypertension (88.9 %), abnormal blood lipids (70.3 %), cardiovascular disease (28.6 %), and diabetic peripheral neuropathy (25.4 %). The other complications rates included the following: diabetic retinopathy (20.7 %), cerebrovascular disease (19.0 %), lower limb angiopathy (14.6 %), renal diseases (11.4 %), and other complications (8.2 %).

One hundred and forty two patients (34.5 %) experienced symptoms of depression (BDI ≥ 14), and the prevalence of co-morbid depression was 5.7 % in this study population; 56 patients had major depression (BDI ≥ 21), and 86 patients had moderate depression (BDI ≥ 14&BDI < 21). The patients with symptoms of depression had lower levels of social support (t = 12.60; *P* < 0.001), and presented poorer glycemic control (χ^2^ = 9.72; *P* = 0.002), a longer duration of diabetes (t = −3.17; *P* = 0.002), and greater number of complications (t = −4.98; *P* = 0.00) as well as had fewer hours of sleep per day (t = 4.38; *P* < 0.001) and fewer exercise sessions per week (t = 3.35; *P* < 0.001), compared with the patients without depression (Table 1). The patients with a family history of diabetes presented a lower prevalence of depression symptoms (24.2 % vs 43.2 %, χ^2^ = 16.42; *P* < 0.001), compared to the patients without a family history of diabetes in this sample (Table 1).

### Risk and protective factors associated with depression

In this study, no significant differences were observed among the patients in different age group diabetes (Table 2). Regarding marital status, being single was a significant risk factor (OR 2.51; 95 % CI: 1.29–4.90; *P* = 0.007) compared with being married. The patients covered by Urban Employees Basic Medical Insurance (UEMI) were more likely to suffer from depression symptoms (OR 2.38; 95 % CI: 1.12–5.04; *P* = 0.024) compared with the patients covered by the Government Employee Insurance Scheme (GIS). Consistent with previous findings, social support was also identified as a protective factor (OR 0.76; 95 % CI: 0.69–0.83; *P* < 0.0001) (Table 2).

**Table 2:** Multivariate logistic regression model predicting co-morbid depression (BDI score ≥ 14) by demographic, clinical, and behavior factors (*n* = 412)

Variables	OR (95 % CI)
Demographic factors	
Men	1.13(0.57,2.24)
Age	
40–59 years vs. <40 years	0.29(0.09,0.97)
≥60 years vs. <40 years	0.58(0.15,2.27)
High school or less	0.94(0.45,1.80)
Being single	2.51(1.29,4.90)
Employment status	
Unemployed vs. employed	4.39(0.89,21.77)
Retired vs. employed	0.74(0.31,1.76)
Insurance	
UEMI vs. GIS	2.38(1.12,5.04)
URMI vs. GIS	0.95(0.40,2.27)
NCMS vs. GIS	0.18(0.19,1.82)
Other insurance vs. GIS	1.64(0.38,7.06)
None insurance vs. GIS	0.77(0.22,2.65)
Social support	0.76(0.69,0.83)
Clinical factors	
HbA1c ≥7.00 %	1.89(1.10,3.53)
BMI ≥25 kg/m^2^	1.12(1.02,1.24)
History of diabtes	0.49(0.26,0.90)
Duration of diabetes	1.11(0.72,1.72)
Diabetes complications	1.12(0.94,1.34)
Oral agents	1.05(0.41,2.73)
Insulin	1.93(0.99,3.82)
Behavior factors	
Smoking currently	1.02(0.45,2.32)
Drinking currently	0.65(0.30,1.42)
Excise times per week	0.92(0.46,1.86)
Sleeping hours per day	0.82(0.67,1.00)

With regard to the clinical characteristics, two risk factors for depression symptoms were identified, including a higher glycated hemoglobin level (HbA1c ≥7.00 %, OR 1.89; 95 % CI: 1.10–3.53; *P* = 0.045), and a higher body-mass index (BMI ≥ 25 kg/m^2^; OR 1.12; 95 % CI: 1.02–1.24; *P* = 0.024). A family history of diabetes was also identified as a protective factor (OR 0.49; 95 % CI: 0.26–0.90; *P* = 0.022) in this study.

## Discussion

Depressive symptoms impede diabetes care tasks and negatively influence health status [61, 62]; furthermore, these depression symptoms not only can’t be identified by the patients, but also can’t be recognized by care providers [61], hence, leading to under-treatment [63]. In this study, we documented that 5.7 % of a study population of type 2 DM patients had symptoms of depression. The prevalence was lower than that reported in India (23.4 % and above) [11, 14–16] and in Baltimore of USA (41.3 %) [12]. Increased risk of depression in patients with diabetes may be attributed to lifestyle and health behaviors, the previous studies suggested the following important factors are related to depression in patients with diabetes: the occurrence of chronic or acute complications, persistent poor glycemic control and the need for insulin therapy [46, 64, 48]. In this study, we found that a higher HbA1c level was a risk factor for the presence of depression symptoms, rather than the number of diabetes complications. Another two studies have also suggested that depression was strongly associated with poor glycemic control and the number of co-morbid conditions [46, 64]. There was a different result from another study conducted by Pouwer F et al [65], which showed that the prevalence of depression increased in patients with type 2 DM and co-morbid chronic diseases, but not in patients with type 2 DM only. In addition, functional limitations that often accompany co-morbid chronic disease could play an essential role in the development of depression in type 2 diabetes [65]. Similarly, a recent study suggested that micro- and macro-vascular diabetic complications were associated with depression [66]. Some researchers noted that this may be resulted from the differences in either the presentation of symptoms or a lack of cultural appropriateness of western methods of identifying depression [22, 17]. Lloyd CE etal found that the variation in symptoms and ways to describe depressive symptomatology can be seen as culturally influenced [7]. However, the mechanism for these differences still remains unclear, and should be further examined in the future. For example, it is important to understand the local term(s) and the culturally distinctive understandings of the causes of depression, the effects of particular health problems, and help-seeking in the community if identification and treatment of co-morbid diabetes and depression is to be improved [22].

In addition, a higher BMI (≥25 kg/m^2^) was also identified as a risk factor for depression symptoms in this study. It was consistent with a previous study [67] which indicated that the BMI contributed to depression indirectly via diabetes-related medical symptoms. Previous study showed that insulin therapy has a significant association with depression among adult patients with diabetes [68], however, insulin therapy wasn’t associated with the depression symptoms in this study, this may be related to the characteristics and the size of this sample.

It’s interesting that a family history of diabetes was identified as a protective factor against symptoms of depression in patients with diabetes in this study. A previous study suggested that an increase in knowledge concerning diabetes care could relieve symptoms of anxiety and depression [69]. A positive family setting provides an opportunity for a patient to obtain personal experience in chronic disease management and consequently manage his/her own disease more effectively [70]. Another protective factor against the presence of depression symptoms was the high social support level in this study. Previous study also confirmed this result, which suggested that if social support level can be strengthened in patients with type 2 DM, psychological factors can be improved [71]. A mediation analysis indicated that the effect of social support on diabetes-related medical symptoms was fully accounted for by social support’s adverse effect on depression [72]. A community-based study on the patients with type 2 DM, in China, showed that high salary and more subjective social support could improve the quality of life in diabetic patients with depressive symptoms [73].

Being single, including being unmarried, widowed, and divorced, was also identified as a risk factor for symptoms of depression, which coincides with the results as reported by José Francisco [46] and M. M. Collins [48]. Being different from the previous studies, which showed that women with or without diabetes experience a higher prevalence of depression than men [62, 64, 48, 74], no significant difference was observed between males and females in this study. The reasons for these differences are unclear and should be further examined in the future, which is most likely connected with the variance of research methods and population characteristics.

In addition, we found that the patients covered by UEMI were more likely to suffer from symptoms of depression compared with the patients covered by GIS. In China, the patients covered by GIS have the highest reimbursement rates, so they had a lighter burden of medical care and less disease-related mental stress than the patients, covered by UEMI, who personally incur high medical expenses.

Type 2 DM is a crucial public health issue in China, with massive medical, social, and economic effects; few studies focus on depression in patients with diabetes. The results of this study should be further confirmed in the future, and should be investigated in different populations, to present a more comprehensive representation of the magnitude of this problem. The incidence of depression in patients with type 2 DM mellitus should be paid more attention.

### Limitations

Our study may be the first to be conducted in China to investigate the prevalence and influencing factors of undiagnosed depression depression in patients with type 2 diabetes mellitus. Systematic random sampling technique, Beck Depression Inventory (BDI), and Social Support Rate Scale (SSRS) were also used in our study. However, our study has a few limitations. First, the samples were recruited from a general hospital and could not represent all of the patients in China. Second, the excluding cases of diagnosed depression in this study also had influence on the prevalence of depression symptoms among the patients with type 2 DM. Third, this study is cross-sectional where causal relationship between diabetes and depression cannot be established. Variables identified as significantly associated with depression may precede depression, but in some cases, these variables could also occur as a result of depression; thus, high BDI scores among patients with diabetes must be interpreted with caution. Longitudinal prospective studies are needed to shed more light on the potential relationship between depression and diabetes mellitus in the future.

## Conclusions

The prevalence of co-morbid depression was 5.7 % among Chinese subjects with type 2 DM in this study. High HbA1c level, high BMI score, being single, and low quality health insurance were associated with the presence of depression. These findings support a recommendation for routine screening in China for depression in patients with diabetes, especially for those in primary care, to reduce the number of the depressed or the misrecognized depressed diabetic patients and consequently offer them a better quality of life.

## Additional file

Additional file 1:
**The characteristic of non-responders in this study.**
