# Supplementary material for: Prevalence and influencing factors of co-morbid depression in patients with type 2 diabetes mellitus: a General Hospital based study
Source: Diabetol Metab Syndr. 2015 Jun 30;7:60. doi: 10.1186/s13098-015-0053-0 (PMC4499190; doi:10.1186/s13098-015-0053-0)
Supplement: Additional file 1: — The characteristic of non-responders in this study. [file 13098_2015_53_MOESM1_ESM.docx]

| **Variables** | **Total sample**  **(n)** |
| --- | --- |
| n(%) | 38 |
| **Demographic factors** |  |
| Men | 18 (47.4) |
| Age (years) |  |
| <40 | 3 ( 7.89) |
| 40-59 | 15 ( 39.47) |
| ≥60 | 20 (52.64) |
| High school or less | 19 (50.00) |
| **Clinical factors** |  |
| History of diabetes | 18 (47.37) |
| Oral agents | 33 (86.84) |
| Insulin | 5 (13.16) |
|  |  |
